# Use of Peptide Nucleic Acids to Manipulate Gene Expression in the Malaria Parasite *Plasmodium falciparum*

**DOI:** 10.1371/journal.pone.0086802

**Published:** 2014-01-22

**Authors:** Netanel Kolevzon, Abed Nasereddin, Shankar Naik, Eylon Yavin, Ron Dzikowski

**Affiliations:** 1 Department of Microbiology and Molecular Genetics, The institute for Medical Research Israel - Canada, The Kuvin Center for the Study of Infectious and Tropical Diseases, The Hebrew University-Hadassah Medical School, Jerusalem, Israel; 2 Institute for Drug Research, School of Pharmacy, Faculty of Medicine, The Hebrew University of Jerusalem, Jerusalem, Israel; Centre National de la Recherche Scientifique, France

## Abstract

One of the major concerns in treating malaria by conventional small drug molecules is the rapid emergence of drug resistance. Specific silencing of essential genes by antisense oliogomers has been proposed as an alternative approach that may result in antimalarial activity which is not associated with drug resistance. In addition, such an approach could be an important biological tool for studying many genes' function by reverse genetics. Here we present a novel methodology of using peptide nucleic acids (PNAs) as a useful tool for gene silencing in *Plasmodium falciparum*. PNAs, designed as specific antisense molecules, were conjugated to a cell penetrating peptide (CPP); namely, octa-D-lysine via the C-terminus, to allow facile delivery through cell membranes. PNAs added to *P. falciparum* cultures were found exclusively in infected erythrocytes and were eventually localized in nuclei of the parasites at all stages of intra erythrocytic development. We show that these PNAs specifically down regulated both a stably expressed transgene as well as an endogenous essential gene, which significantly reduced parasites' viability. This study paves the way for a simple approach to silence a variety of *P. falciparum* genes as means of deciphering their function and potentially to develop highly specific and potent antimalarial agents.

## Introduction

Malaria is one of the major infectious diseases influencing human kind today. The causative agent of the deadliest form of malaria in humans is the protozoan parasite *Plasmodium falciparum*. This parasite is estimated to infect 300–600 million people worldwide each year, resulting in 1–3 million deaths, primarily of young children and pregnant women [1]. *P. falciparum* replicates within the circulating red blood cells of an infected individual, and its virulence is attributed to the ability of the parasites to modify the erythrocyte surface and to evade the host immune attack. Parasite populations have developed resistance to almost every drug used to treat malaria, including drugs acting at different stages in the complex life cycle of this parasite [2]. In view of the absence of an effective vaccine and the rapid evolution of drug resistance, new approaches are needed in order to fight the disease.

Even though the genome of *P. falciparum* was entirely sequenced more than a decade ago [3] approximately half of its ∼ 5700 genes remained with unknown function. This is mainly due to the lack of genetic tools that will allow rapid application of reverse genetics approaches. The genomes of Plasmodium parasites lack genes encoding components of the RNAi machinery [4] and techniques for genetic disruption in Plasmodium are applicable only in elucidating the function of genes that are not essential for parasite development, while genetic deletion of essential genes is lethal. Recently, new techniques have been developed that allow controlled inducible manipulation of protein expression [5], [6]. However, creation of knocked-in transgenic lines remains a pre-requisite for successful application of these tools and requires much effort and time.

Interestingly, the genome of *P. falciparum* has approximately 80% AT bp and is one of the most AT-rich genomes [3]. This substantial difference from the human genome opens the opportunity of targeting the parasite’s genome by sequence specific inhibitors, namely, antisense oligonucleotides (ASO). Such ASOs could be highly specific to a variety of essential mRNA targets of the parasite, resulting in drug candidates that are (1) less toxic, (2) highly specific, and (3) easily combined to target several genes for higher efficacy. Nonetheless, several hurdles exist before such an approach may be realized. These include cellular uptake into infected erythrocytes, serum stability, low or no off-target effects, and high potency.

Since the early 1990s several studies using ASO that target a variety of genes in *P. falciparum* were reported. Using metabolically stable phosphothioated (PS) ASO [7], [8], [9], [10], sequence-specific down-regulation of several endogenous genes was shown at concentrations of ASO typically in the range of 0.1 to 0.5 µM. However, non-specific growth inhibition was observed at higher ASO concentrations. This was correlated with the inhibition of merozoite invasion of red blood cells as a consequence of the anionic nature of the PS-ASO [11], [12].

In recent years, the use of nanoparticles (NPs) as ASO delivery vehicles has been examined as means of improving the potency of ASO while lowering non-specific interactions. We decided to explore the antisense activity of peptide nucleic acids (PNAs). PNA is a DNA mimic that efficiently hybridizes to complementary RNA and is metabolically stable [13]. Having a neutral backbone we speculated that such molecules would not have delivery issues that have been found in negatively charged ASO. In addition, as PNAs are easily modified with cationic cell penetrating peptides (CPPs), we synthesized peptide-PNA conjugates as cell-permeable molecules and studied their gene-silencing activity in blood stages of *P. falciparum*. We show that antisense PNA molecules can be used as an efficient tool to manipulate gene expression in *P. falciparum.* Further, targeting expression of a housekeeping gene significantly reduced parasite viability, providing proof of principal for the use of PNAs as a novel tool for studying gene function in *Plasmodium* In addition, improvement in PNA synthesis which will reduce production cost would potentially pave the way for using it as a new therapeutic agent for treating malaria.

## Materials and Methods

### Cell cultures

All parasites used were derivatives of the NF54 parasite line and were cultivated at 5% haematocrit in RPMI 1640 medium, 0.5% Albumax II (Invitrogen), 0.25% sodium bicarbonate and 0.1 mg/ml gentamicin. Parasites were incubated at 37°C in an atmosphere of 5% oxygen, 5% carbon dioxide and 90% nitrogen. For the experiments presented in Fig. S4B, parasite cultures were synchronized using percoll/sorbitol gradient centrifugation as previously described [14], [15]. Briefly, infected RBCs were layered on a step gradient of 40%/70% percoll containing 6% sorbitol. The gradients were then centrifuged at 12000 *g* for 20 min at room temperature. Highly synchronized, late stage parasites were recovered from the 40%/70% interphase, washed twice with complete culture media and placed back in culture. The level of parasitemia was calculated by counting 3 independent blood smears stained with Giemsa under light microscope. Blood was anonymously donated from the blood bank of Hadassah Medical Center (http://www.hadassah-med.com/).

### Luciferase Assays

Luciferase activity was measured from 200 µl of culture containing tightly synchronized ring stage parasite. Infected red blood cells (iRBC) were pelleted by centrifugation and lysed in 50 µl Glo Lysis Buffer® (Promega). Luciferase activity was measured immediately after adding 100 µl Bright-Glo® luciferase reagent (Promega) in FLUOROSKAN FL luminometer (Thermo). The luciferase activity of each clonal cell line was determined in at least three independent experiments and was normalized to 1% parasitemia.

### In-vivo and fixed parasite imaging

For *in vivo* imaging cultures containing approximately 1% parasitemia were incubated with PNAs. Unsynchronized parasites were pelleted, diluted with PBS x 1 and incubated with Hoechst 33342 Fluorescent Stain (Thermo Scientific). Then samples were laid on a tephlon coated slide (EMS), covered with 18×18 cover slides and immediately visualized. For quantification, parasites were isolated from RBCs by saponin lysis as described below and fixed with 5% PFA. Images were taken using Apochromat oil immersion objective with x100 magnification (Olympus) on an Olympus IX71S8F microscope equipped with Exi Blue™ Fast camera (QImaging).

### SDS-PAGE and Western blot analysis

To collect parasite proteins, iRBCs were lysed with 5% saponin on ice. Parasites were washed with PBSx1 and re-suspended with 2 x Lameli sample buffer (Sigma). Proteins were loaded on 4–20% Polyacrylamide gels (Bio-Rad) along with protein size marker (Precision plus, Bio-Rad) and were subjected to SDS-PAGE at 100 volts for 1 hour. Proteins were electroblotted to nitrocellulose membrane (Schleicher & Schuell, BioScience GmbH) using a wet transfer apparatus (Bio-Rad) at 135 mA for 90 minutes. Membranes were blocked with 5% skim milk (Difco) in PBST for 1 hour at RT. Immunodetection was carried out by incubating the membrane with a primary antibody diluted with blocking solution as follows: 1;1000 Mouse α-HA (monoclonal, Roche); 1∶500 rabbit α-Pf39 (polyclonal, MR4); 1∶1000 rabbit α-aldolase (polyclonal, kindly given by Dr. Jake Baum, WEHI) followed by incubation with rabbit α-mouse or mouse α-rabbit secondary antibodies (1∶10000) conjugated to Horseradish Peroxidase (HRP) (Jackson, ImmunnoResearch Laboratories, INC). Membranes were developed by EZ/ECL solution (Israel Biological Industries, LTD).

### Real-time RT-qPCR

RNA extraction and cDNA synthesis was performed as described [16]. Briefly, RNA was extracted with the TRIZOL LS Reagent® as described [17] and purified on PureLink column (Invitrogen) according to manufacturer’s protocol. Isolated RNA was then treated with Deoxyribonuclease I® (Invitrogen) to degrade contaminating gDNA. cDNA synthesis was performed from 800 ng total RNA with Superscript II Rnase H reverse transcriptase ® (Invitrogen) with random primers ® (Invitrogen) as described by the manufacturer. For RT-qPCR reactions to detect luciferase transcription we used luciferase primers sets published earlier [18]. Transcript copy numbers were determined using the formula 2^−ΔΔCT^ as described in the Applied Biosystems User Bulletin 2 using NF54 gDNA as the calibrator. Specifically, relative copy number was calculated as 2 exponential negative ((Ct target gene in cDNA – Ct reference gene in cDNA)-(Ct target gene in gDNA-Ct target gene in gDNA)). The housekeeping genes arginyl-tRNA synthetase (PFL0900c), P61-fructose bisphosphate aldolase (PF14_0425) and glutaminyl-tRNA synthetase (PF13_0170) were used as control genes in all RT-qPCR assays as described [18]. The reference gene used for result presentations in the presented graphs presented in the manuscript is arginyl-tRNA synthetase. All RT-qPCR assays were performed in triplicate for each template with no apparent differences, and the experiment was completed three times in its entirety, again with no significant differences.

### PNA Synthesis

Thiazole orange modified PNA monomer was synthesized as previously reported [19] with the following slight modification: To improve coupling of TO-CH_2_COOH to PNA backbone, the acid (TO-CH_2_-COOH, 350 mg, 1 mmol) was activated with 2-(1H-7-Azabenzotriazol-1-yl)--1,1,3,3-tetramethyl uronium hexafluorophosphate Methanaminium (HATU, 419 mg, 1.1 mmol), hydroxybenzotrilazole (HOBT, 149 mg, 1.1 mmol), and diisopropylethylamine (DIPEA, 0.384 mL, 2.2 mmol) and the solution (3 mL in dry DMF) was added to the PNA backbone (302 mg, 1.1 mmols, dissolved in 2 ml of dry DMF). The reaction was allowed to proceed for 6 hours at 55°C. Column chromatography (95:5 dichloromethane:methanol) of crude product afforded (424 mg, 0.7 mmol) of desired material (70% yield). Subsequent synthetic steps (Boc/tBu ester deprotection and Fmoc addition to alpha amine) were performed as previously reported [19]. The synthesis of PNAs was carried out on a TGA-NovaSyn PEG (Merck) resin at a 18 µmols scale per PNA sequence using alpha-amino Fmoc protected PNA monomers (PolyOrg, Inc., USA) and Fmoc-D-Lys(BOC)-OH (Chem Impex, USA) as previously described [19]. The first monomer (Fmoc-D-Lys-(Boc)) was coupled to the free hydroxyl groups of the resin using 10eq. of the amino acid, 5 eq. of diisopropylcarbodiimide (DIC), and 0.1 eq of 4-dimethylaminopyridine (DMAP) in dry DMF. All PNAs were HPLC purified (Luna C18, 10 microns, 100Å, 250×21.2 mm Phenomenex column) and analyzed by Maldi-TOF MS (See **Table 1**). HPLC chromatograms of PNAs are provided in (**Fig. S1)**.

**Table 1:** PNA sequences and their corresponding Maldi-TOF MS assignments.

Target	Sequence	Mass calc.	Mass found	Abbr.
Luciferase	COOH-(dK)_8_GACCTTCT(TO)CCTTGGCG(TR)	6108	6135	Luc-PNA
Luciferase	COOH-(dK)_8_GCTCTGCT(TO)GCGCCTAT	5753	5743	Scr-Luc-PNA
PfSec-13	COOH-(dK)_8_TGGATAGT(TO)CCTTCTAG	5815	5856	Sec13-PNA
PfSec-13	COOH-(dK)_8_GGATCTCTGT(TO)TATGCA	5815	5835	Scr-Sec13-PNA
AG-Sequence	COOH-(dK)_8_AAAGGGAAAGGAAA-TO	5550	5557	AG-PNA

## Results

**Figure 1:**
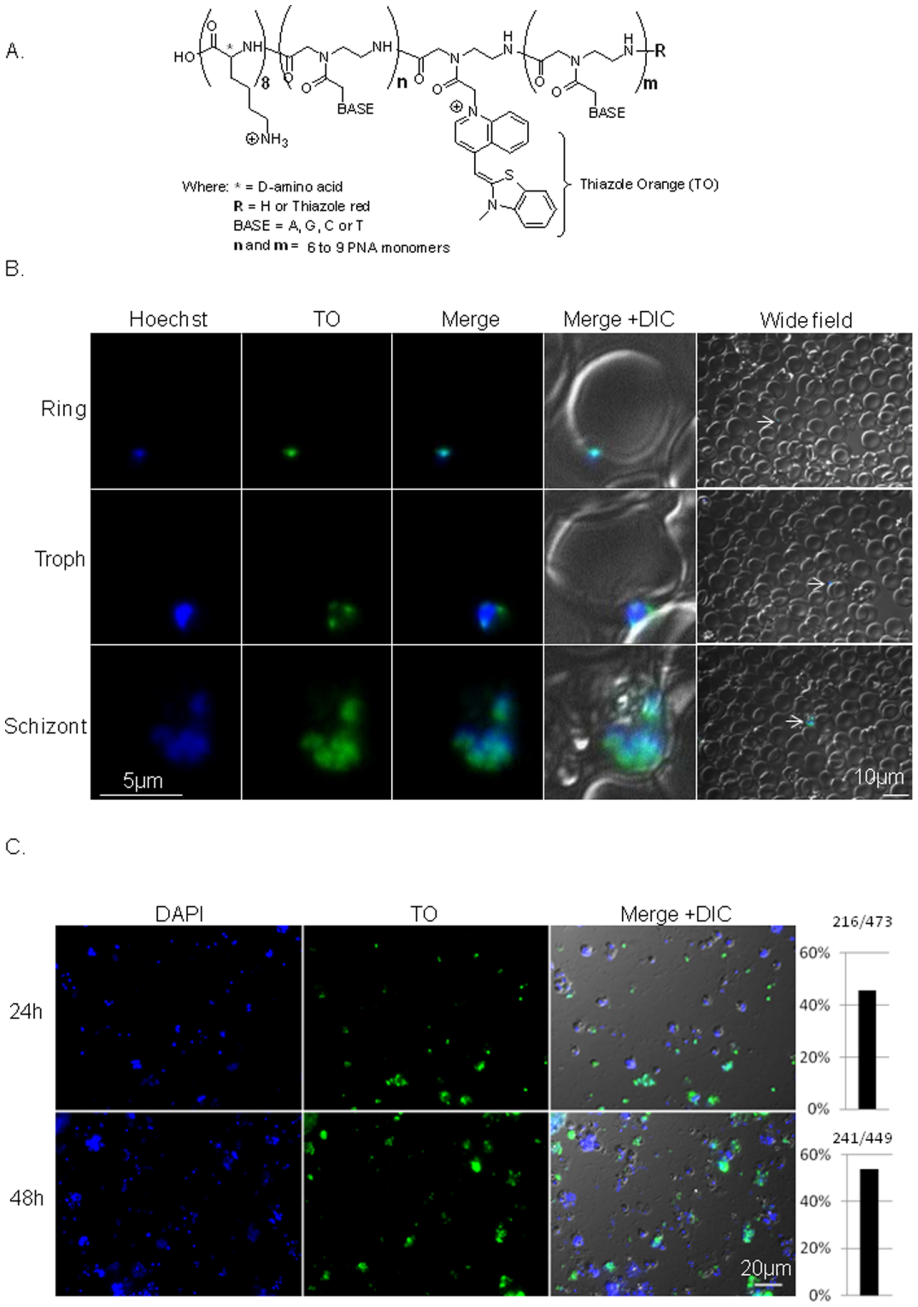
PNA is targeted to the nuclei of intra-erythrocytic plasmodium parasites . **(A)**, Schematics of the PNAs molecules. **(B)**, *In vivo* imaging of Luc-PNA in infected RBCs 96h after addition of 0.6 µM Luc-PNA into unsynchronized parasites' culture. The PNAs were included in the culture media for the initial 24h, after which the culture media was replaced daily without PNAs. The Luc-PNA is seen in nuclei of parasites in various stages of the IDC. Arrows in the wide field point to the iRBC presented in high magnification. Images were taken using exposure time of at least 700 ms. (C), Quantification of PNA molecules observed in parasites 24h (N = 473) and 48h (N = 449) after incubation into the culture media. The percentage of parasites in which the PNAs could be observed is presented on the right columns. Images were taken using exposure time of 40 ms.

A prerequisite for using PNAs as a tool to manipulate gene expression in Plasmodium is the ability of these molecules to reach and hybridize to the parasites' complementary RNA. In the intracellular blood stages of *P. falciparum* this is a challenging since the PNAs have to traverse three membranes before they reach the parasite: the erythrocyte membrane, the parasitophorous vacuole, and the parasites' plasma membrane prior to their delivery into the nucleus. Therefore, a stretch of eight D-lysines were conjugated to the C-terminus of the PNA molecule for improving the molecule’s water solubility and cell permeability. We chose the D amino acid as means of improving stability to peptidases (**Fig. 1A** **&** **Table 1**).

As a first step to determine if PNAs can reach RNA of blood stage parasites and influence gene expression we used NF54-*luc* parasites [20]. In this transgenic parasite line the firefly *luciferase* gene was integrated into the genome and constitutively expressed by the *hrp2* promoter. Specific antisense PNAs were designed to bind only *luciferase* RNA and no other sequence in the genome. To enable visualization of the PNA molecules in these initial experiments, they were conjugated to two fluorescent markers: Thiazole orange (TO) dye replacing one nucleotide (A) in the middle of the PNA sequence and thiazole red (TR) at the 3' (**Fig. 1A** **, Fig. S1 &** **Table 1**). This design was used in order to follow PNA uptake into erythrocytes at 2 different wavelengths. The TO probe (λexc  =  500 nm) can serve as a surrogate base that upon hybridization to complementary RNA is expected to increase its fluorescence [19], [21], [22], [23]. The TR probe is expected to be continuously fluorescent at a different wavelength (λexc  =  630 nm) independent of hybridization. Parasites were cultured in the presence of 0.6 µM of the designed PNAs for the initial 24 hrs of the experiment, after which the parasites were maintained in standard culture media. Individual parasites were visualized with fluorescence microscopy *in vivo* at 24h, 48h, 72h, and 96h after the initiation of the experiment. Interestingly, 24h post incubation TO signal could already be detected in parasite at various stages of development, where in late stages it seems to be concentrated in the FV (Fig S2A). At 48h post incubation PNA signals could already be detected in the parasites' nucleus (Fig. S2B). The Luc-PNA molecules localized to the nucleus of parasites at various stages of intra-erythrocytic development (IDC), even 96h post incubation (**Fig. 1B**). The presence of PNAs in ring stages 96h post incubation indicate that some of the PNA molecules are maintained during schizogony in the nuclei of the daughter cells as seen also in schizonts. This data is the first evidence that PNA molecules added to culture media are targeted to Plasmodium nuclei.

In addition to the *in vivo* imaging, we isolated parasites after incubation with Luc-PNA by lysing the iRBC and fixing them on slides. We were able to visualize the Luc-PNAs in approximately 50% of the parasites when they were incubated in the culture media for 24h and 48h (**Fig. 1C**) indicating the presence of the PNAs in at least 50% of the parasites at this time point.

**Figure 2:**
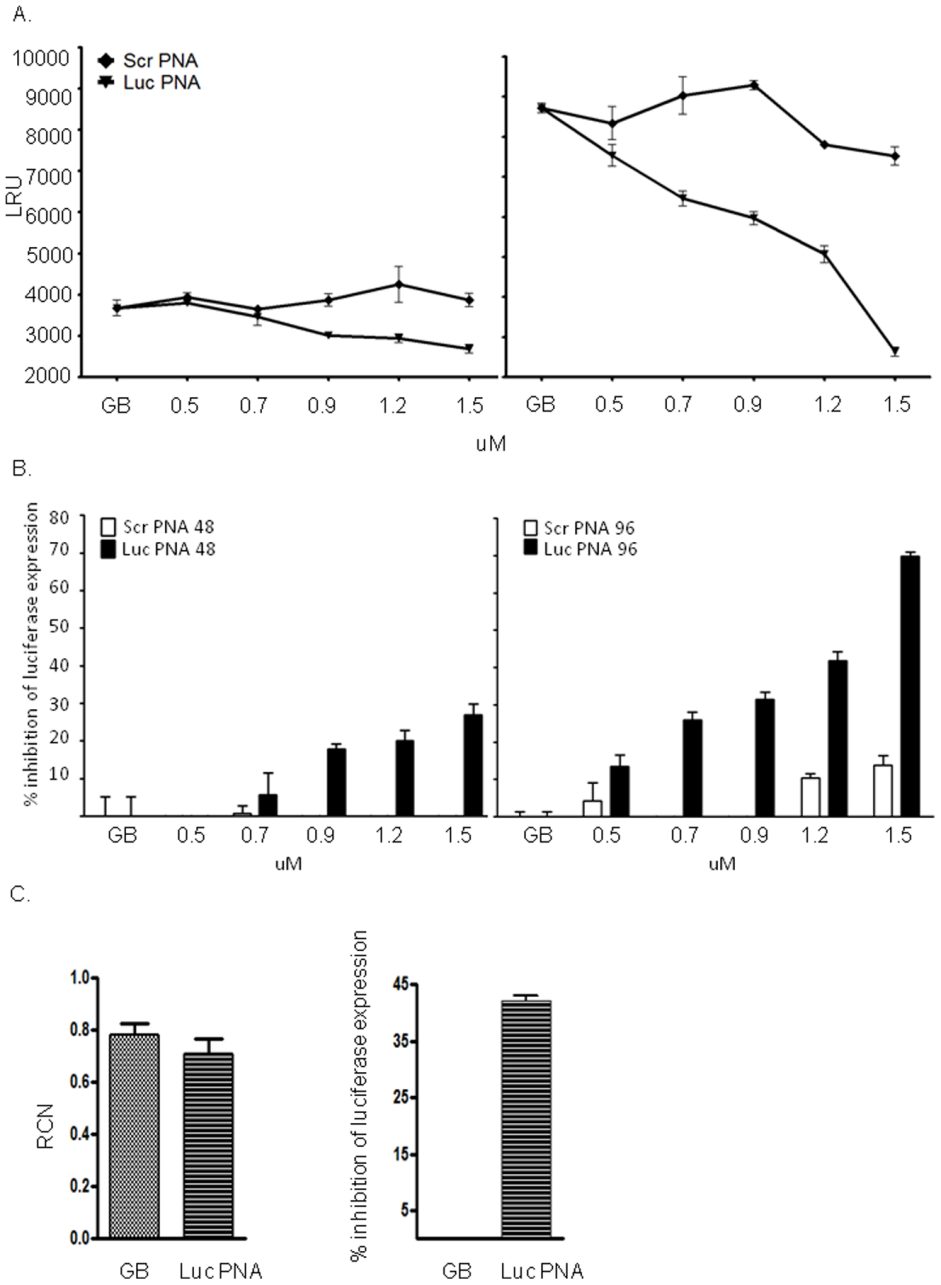
Specific inhibition of gene expression using PNAs in transgenic *P. falciparum* parasites. Dose dependent inhibition of expression of *luciferase* reporter gene in NF54-luc (GB). These parasites constitutively express *luciferase* from a chromosomal locus [20]. Increasing concentrations of 0.5, 0.7, 0.9, 1.2, and 1.5 µM Luc-PNA were added to the culture media as well as similar concentrations of the scrambled PNA sequence (Scr-Luc-PNA). Parasites were incubated with the PNA molecules for 48h (left panel), then washed and cultured for an additional 48h (96h post incubation, right panel) in the absence of PNA in the media. Specific dose dependent inhibition of *luciferase* expression was found to be more robust after 96h **(A)**, reaching up to 70% inhibition **(B).** LRU, luminescence rate units. ScrPNA, Scr-Luc-PNA. **(C).** PNA treatment down regulate luciferase expression but does influence the levels of steady state mRNA. Left panel: steady state mRNA levels of luciferase in GB parasites treated with 1.2 µM LucPNA vs untreated parasites (GB). RCN, Relative copy number. Right panel: inhibition of luciferase expression following incubation with 1.2 µM LucPNA for 48h. All experiments were done in triplicate and the average is presented with SE.

Encouraged by the fact that our PNAs can reach the parasites' nucleus, we then examined how they influenced *luciferase* activity. We performed *luciferase* assays on un-synchronized parasites incubated with increasing concentrations of Luc-PNA and compared it with parasites that were incubated with scrambled PNA (Scr-Luc-PNA) which has no sequence similarity in the *P. falciparum* genome. Parasites were incubated with the PNAs in 96-wells plate for 48h, after which the media was exchanged daily for additional 48h (altogether 96h). After 96h, parasites in all treatments reached similar parasitemia of ∼ 4%. We found that incubation with the Luc-PNA had a specific dose dependent inhibition effect on *luciferase* expression. Interestingly, even though the media was exchanged after 48h the inhibition effect on *luciferase* expression had increased a generation later reaching up to ∼ 70% inhibition at 1.5 µM (**Fig. 2A, B**). No inhibition was observed in parasites incubated with increasing concentrations of non-specific Scr-Luc-PNA molecules indicating that the PNA specifically down regulated the expression of the gene it was designed to. Interestingly we found that the decrease in luciferase expression was not accompanied with detectable changes in the levels of its steady state mRNA levels. This implies that the mechanism by which PNAs down regulate genes in *P. falciparum* is post transcription. In addition, as PNAs do not evoke RNAse H activity when bound to target RNA, it is expected that RNA levels would not change.

**Figure 3:**
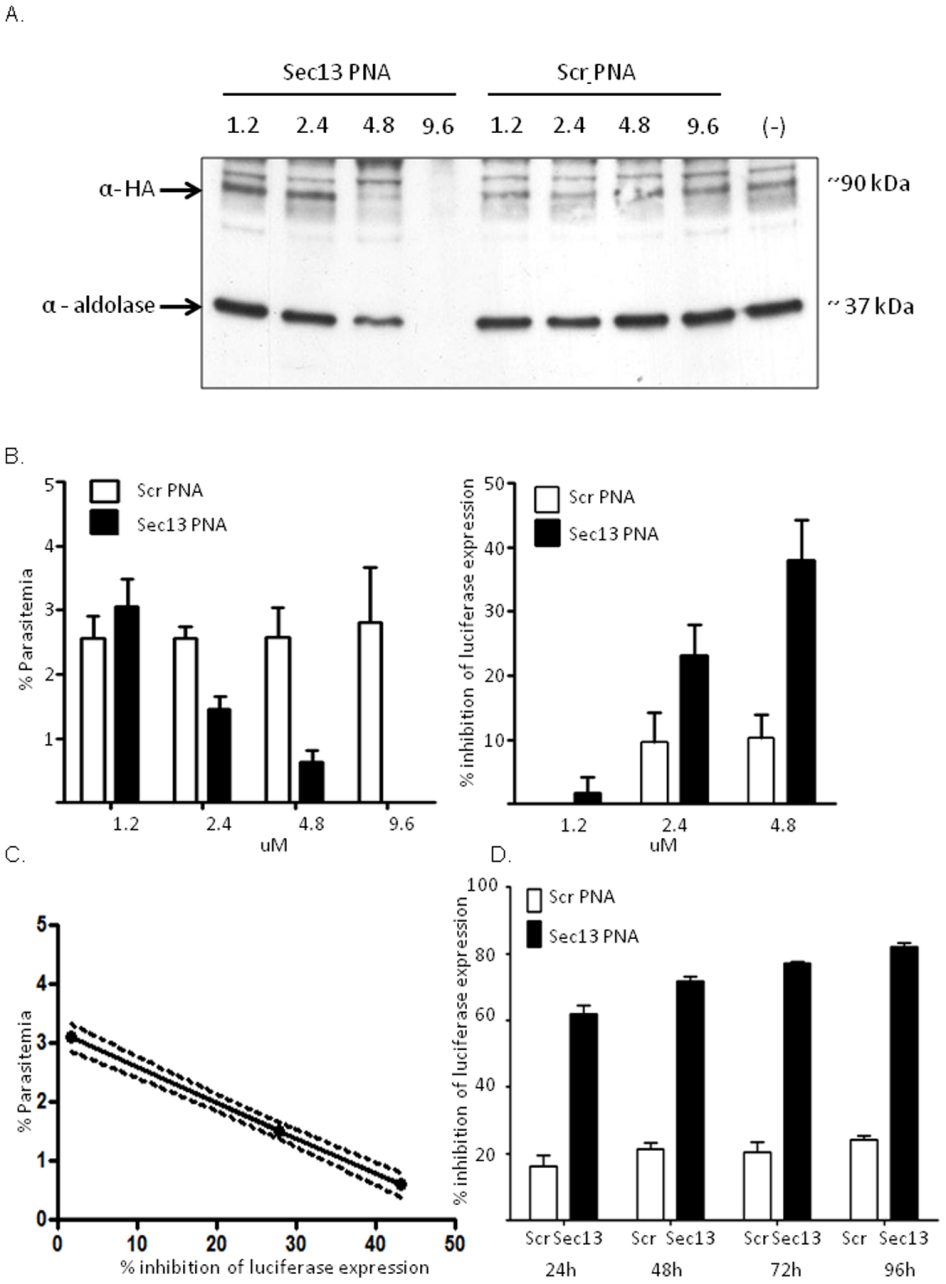
Specific down regulation of endogenous gene in *P. falciparum* using PNA molecules. **(A)**, Specific dose dependent down-regulation of PfSec13 expression in parasites incubated with 1.2, 2.4, 4.8 and 9.6 µM Sec13-PNA compared with scrambled PNA sequence (Scr-Sec13-PNA) observed by western blot analysis using antibodies against endogenously tagged PfSec13 (α-HA) and aldolase. (–), no treatment. ScrPNA, Scr-Sec13-PNA. **(B)**, Down-regulation of an essential gene using specific Sec13-PNA molecules reduces parasites' viability. NF54-*luc* parasites constitutively expressing the *luciferase* reporter gene were incubated with 1.2, 2.4, 4.8 and 9.6 µM of either Sec13-PNA or Scr-Sec13-PNA. 72h post incubation the parasitemia was counted by direct microscopy (left panel) as well as inhibition of *luciferase* expression (right panel). The parasitemia decreased in a dose dependent manner only in parasite treated with Sec13-PNA. The gradual increase in inhibition of parasites' viability by Sec13-PNA was calculated as the percentage of *luciferase* reads compared with untreated NF54-*luc* parasites **(C).** The inhibition in growth is correlated with decrease in luciferase expression in parasite treated with Sec13-PNA. R^2^ = 0.99, P<0.05. Dashed line represents 95% confidence interval slope **(D)**. Over time inhibition of parasites viability using PNA. Parasites were incubated with 4.8 µM Sec13-PNA or Scr-Sec13-PNA and luciferase expression was measured 24h, 48h, 72h, and 96h post incubation. The specific reduction in parasites viability is presented as percentage of inhibition of *lucifearse* expression compared with untreated NF54-*luc* parasites as above. All experiments were done in triplicate and the average is presented with SE.

The ability to down-regulate the *luciferase* transgene provided the first evidence that PNAs can be used as a useful tool to manipulate gene expression in *Plasmodium*. We were also interested to examine their use on expression of an endogenous gene which is essential for parasite proliferation in human RBCs and examine the ability of PNAs to eliminate infection. As an endogenous target gene we chose PfSec13 (PF3D7_1230700), which is a plasmodium homologue to Sec13, a core component of the nuclear pore complex as well as the COPII-coated vesicle transport system in the cytoplasm [24]. We created a parasite line in which PfSec13 is endogenously tagged with an HA epitope [25] which allows us to detect protein expression even from a small volume of parasites. Specific Sec13-PNA designed to hybridize to PfSec13 mRNA (**Table 1** **, Fig. S1**) were synthesized using the TO base surrogate as a fluorescent marker. After confirming that both TO (**Fig. 1B**) and TR (data not shown) fluorescence signals could be visualized in the first set of PNAs we used only TO in this set of PNA molecules. We have recently showed that these PNAs specifically down regulate PfSec13 and reduce parasites viability compared with an unrelated control sequence (AG-PNA). In order to establish this methodology and determine if these PNAs can eliminate parasites from culture we performed a dose response measurement of PfSec13 expression after incubation with increasing concentrations of the Sec13-PNA. Parasites were incubated with 1.2 µM, 2.4 µM, 4.8 µM and 9.6 µM of either specific PfSec13 PNA (Sec13-PNA) or non-specific scrambled PNA (Scr-Sec13-PNA) for 48h after which the media was exchanged without addition of another dose of PNAs. 72h post incubation parasites reached the parasitemia needed for protein detection by western blot. We found that a dose dependent decrease in protein expression of PfSec13 could already be detected after 48h (**Fig. S3**), nonetheless, this decrease became more robust 72h post incubation where no protein could be detected at the highest concentration of 9.6 µM (**Fig. 3A**). As in the *luciferase* transgene, we did not observe non specific knock-down of protein expression when using the scrambled PNA (**Fig. 3A**) or another non-specific PNA (AG-PNA, **Fig. S3**). In addition we observed no hemolytic effect of the PNA molecules at all concentrations tested (**Fig. S4A**).

In other eukaryotes, Sec13 was found to be an essential protein and attempts to create genetic deletions were found to be lethal [24], [26] and decrease in PfSec13 expression adversely affects parasite viability [25]. To demonstrate that by targeting a plasmodium essential gene we can eliminate parasite from culture we used the NF54-*luc* parasites described above to perform a *luciferase*-based viability assay on parasites exposed to increasing concentration of Sec13-PNA vs. those incubated with Scr-Sec13-PNA. Interestingly, at low concentration of 1.2 µM of Sec13-PNA changes in protein expression could already be detected after 48h but the decrease in *luciferase* expression, which reflects the decrease in viability, was observed only a generation later at 96h post incubation [25]. Therefore, we incubated the PNAs in culture for 48h, and then changed media and measures viability 96h post incubation. To support the *luciferase* assay, Giemsa stained blood smears were made for each treatment and the parasitemia was measured by direct microscopy. Exposure of parasite cultures to increasing concentrations of Sec13-PNA resulted in clear dose dependent inhibition in parasites' proliferation, while no such decrease in parasitemia was found in those that were treated with Scr-Sec13-PNA (**Fig. 3B, left panel**). Strikingly, no live parasites were found in the culture incubated with 9.6 µM Sec13-PNA. Similarly, the level of inhibition in *luciferase* expression compared to untreated NF54-*luc* parasites increased in a dose dependent manner in parasites treated only with the Sec13-PNA (**Fig. 3B, right panel**). The decrease in the parasitemia measured by direct microscopy was tightly correlated with the inhibition in luciferase expression in our viability assays (Fig. 3C). We were further interested to test the inhibition effect of the PNA on parasite viability over time. NF54-*luc* parasite were incubated with the effective dose of 4.8 µM Sec13-PNA and *luciferase* viability assay was performed every 24h for 96h. We found that even though media was exchanged after 48h without addition of fresh PNAs, there was a gradual increase in inhibition in parasites growth over time (**Fig 3D**). Altogether these data suggests that antisense PNA molecules can be used as an efficient tool to down regulate gene expression in blood stages of *P. falciparum* and that targeting essential genes could eliminate parasites from human RBCs *in vitro*.

## Discussion

Specific RNA targeting using antisense PNA molecules is an efficient novel approach to study gene function in Plasmodium, which offers opportunities to develop novel therapeutic approaches to treat malaria. We demonstrated that antisense PNA conjugates with a simple octa-D-lysine CPP successfully and specifically down-regulate *P. falciparum* gene expression. Interestingly, we visualized these PNAs exclusively in iRBCs, initially in the parasites' FV and eventually targeted into the nuclei of parasites at all stages of the cell cycle. The accumulation of the PNA molecules in parasites nuclei suggests that they already hybridize pre-mRNA rather than mature mRNA. In addition, the fact that they do not change the level of steady state mRNA (Fig 2C) points towards post-transcription mode of action possibly by preventing export from the nucleus or blocking translation by steric block of the RNA without the involvement of RNAse H degradation of the mRNA [27]. This efficient specific uptake of PNA molecules by *P. falciparum* could be related to some of the modifications the parasites induce in iRBCs. These parasites express specific proteins that form anion channels on the surface of iRBCs which allow them to take up ions and nutrients from the serum [28], [29]. This could also explain why PNA molecules incubated with parasite cultures at different stages of development had different down regulation efficiency (**Fig. S4B**). In synchronized parasite cultures the antisense activity was more profound when PNAs were added in the trophozoite stage. This could be due to superior uptake of PNAs through the parasite-expressed channels induced by trophozoite-infected RBCs when compared with RBCs infected by ring stage parasites. Nonetheless, the detection of fluorescent PNA signals in approximately 50% of parasites at the early time points after incubation is in agreement the lower down-regulation effect of the PNAs at these time point compared with the later time point. In order to reach the parasite nucleus PNA molecules have to cross several membranes. The efficient delivery of these PNAs through the parasitophorous vacuole, parasites' membrane, and nuclear envelope into the nucleus, could be explained by the eight positive charges originating from the CPP. Interestingly, similar concentrations of different PNAs had different effects on each of the genes used in this study. A lower dose of 1.5 µM Luc-PNA was sufficient to down regulate *luciferase* expression by ∼ 75% while 4.8 µM Sec13-PNA was needed to reach similar decrease in PfSec13 expression. These differences might be related to the nature of the protein investigated. One possible explanation for these differences is that PfSec13, which is an integral component of nuclear pore complex and a key player in COPII-coated vesicles trafficking machinery, has a relatively slow turnover as was recently demonstrated for other scaffold nucleoporins [30]. The expected slow turnover of PfSec13 could explain the overall lower levels of its decreased expression when compared with those observed with *luciferase*. The effect of antisense PNAs designed to target PfSec13 down regulate only *de novo* protein synthesis, while some of the protein could remain in the parasite from earlier cell cycles. This could also be the reason why the decrease in viability as a result of PfSec13 down regulation is observed a generation after the decrease in protein expression levels could be detected.

Over the past decade antisense oligonucleotides targeting different genes were shown to inhibit *in vitro P. falciparum* growth [8], [10], [31], [32] and therefore have been considered as a potential therapeutic strategy against malaria. However, antisense-based therapy was thus far limited by the rapid degradation of the nucleotides *in vivo* as well as their inefficient delivery across cell membranes and their inability to reach their target mRNAs due to their hydrophilic character and high molecular structure [33]. In order to improve stability and to increase intracellular penetration, Föger and co-workers [34] formulated topoisomerase II ASO into biocompatible chitosan based nanoparticles (NPs). They showed that using these NPs they can increase growth inhibition without increasing the hemolytic activity of the ASO on RBCs. However, over 50% growth inhibition was observed when using sense oligos to the same gene, indicating a significant non-specific effect. Similar non-specific effects were reported in a recent study, using cationic nanoemulsions (NE) for the delivery of ASO into infected RBCs [35]. Although high growth inhibition was found for NE/PS-ASO targeting the topoisomerase II gene (ca. 80%), the sense strand showed a significant inhibition in parasite proliferation (ca. 60%); suggesting other mechanisms of action that are not related to antisense activity. However, in our current study non-specific off target effects have not been observed using scrambled PNAs. In addition, an important advantage in the use of CPP-PNA conjugates is in its simplicity. No delivery system is required and the molecule is simply added to cell culture and as we demonstrate they are highly specific. The addition of a delivery system may lead to complications as non-related effects (such as cytotoxicity to healthy cells or off-target effects) may emerge. This highlights the advantage of using DNA analogs that have a neutral backbone and that can readily penetrate cells by the simple addition of a CPP.

Similar to the CPP-PNA conjugate, transductive peptides attached to the 3′ ends of antisense Phosphorodiamidate morpholino oligomers (PMO) allow the oligomers to readily enter cells by crossing multiple membrane barriers. PMOs also inhibit gene expression in a sequence-specific manner and have been recently used as an antisense knock down approach to down-regulate gene expression of the Apicomplexan parasites *Toxoplasma gondii* [36]. In *P. falciparum*, peptide-morpholino oligomers (PMOs) conjugated to an external guide sequence (EGF) RNA have been used to selectively cleave mRNA by targeting RNase P to the gyrase mRNA (PfGyrA) [37]. Similar to our study, these morpholinos were added without the need of a delivery system and were shown to accumulate only in infected RBCs. It would be interesting to directly compare the efficiency of PMOs and PNAs on gene expression of Apicomplxan parasites.

As PNAs are routinely synthesized in Chemistry labs (monomers are commercially available and synthesis is straightforward), this approach can be easily translated to other academic laboratories. This opens the opportunity to explore hundreds of *P. falciparum* genes leading to a better understanding of the Parasite’s biology with the possibility of advancing new drug candidates based on PNA chemistry to the clinic.

## Supporting Information

Figures S1 **HPLC chromatograms of PNA sequences.** PNAs were HPLC purified on a Luna C18 phenomenex column (10 microns, 250×21.2 mm), monitored at 260 nm, using the following eluents: 0.1% TFA in water and acetonitrile; 10-25% gradient of acetonitrile in 30 minutes.
(TIF)

Figure S2 **LucPNAs in targeted to intraerythrocytic parasites 24h and 48h post incubation. (A),** 24h post incubation with 1.2 µM LucPNA the molecules could already be observed in various stages of development particularly in the FV. We could not detect fluorescent signal in ring stages at this time point. **(B)**, 48h after incubation with 1.2 µM LucPNA the molecules already reach parasites nuclei in various stages of development. Images were taken using exposure time of at least 960ms.
(TIF)

Figure S3 **Specific down regulation of endogenous gene in** ***P. falciparum*** **using PNA molecules. (A)**, Specific dose dependent down-regulation of PfSec13 expression in parasites incubated with 1.2, 2.4, and 4.8 µM Sec13-PNA observed by western blot analysis. **(B)**, Down-regulation of an essential gene using specific PNA molecules reduces parasites' viability. NF54-*luc* parasites constitutively expressing the *luciferase* reporter gene were incubated with 1.2, 2.4, or 4.8 µM of either Sec13-PNA or GA-PNA (control). 96h post incubation *luciferase* expression decreased in a dose dependent manner only in parasite treated with Sec13-PNA. The gradual increase in inhibition of parasites' viability by Sec13-PNA is presented in **(C)**. All experiments were done in triplicate and the average is presented with SE.
(TIF)

Figures S4 **PNA incubation causes no hemolysis of RBCs but affect gene expression in a stage dependent manner. (A),** The PNA molecules tested do not cause hemolysis. Optical density (OD) of media containing RBCs incubated with increasing concentrations of Sec13-PNA for 72h were measures at 560 nm in microplate luminometer (Thermo, Fluroskan). Data presented as average of 3 replicates. Error bar represents stdev. **(B),** Stage dependent down regulation of *luciferase* expression using PNAs. Tightly synchronized NF54-*luc* parasites constitutively expressing the *luciferase* reporter gene were incubated for 24h with of 0.6 µM Luc-PNA and GA-PNA (Control) at early (0-16 hpi) and late (18-42 hpi) stage of IDC. Specific stage dependent inhibition of *luciferase* expression was observed in parasites incubated with Luc-PNA 48h post incubation. All experiments were done in triplicate and the average is presented with SE. Negative control of untreated parasites is marked with (-).
(TIF)
