# Supplementary figures and images for: Use of Peptide Nucleic Acids to Manipulate Gene Expression in the Malaria Parasite Plasmodium falciparum
Source: PLoS One. 2014 Jan 22;9(1):e86802. doi: 10.1371/journal.pone.0086802 (PMC3899306; doi:10.1371/journal.pone.0086802)

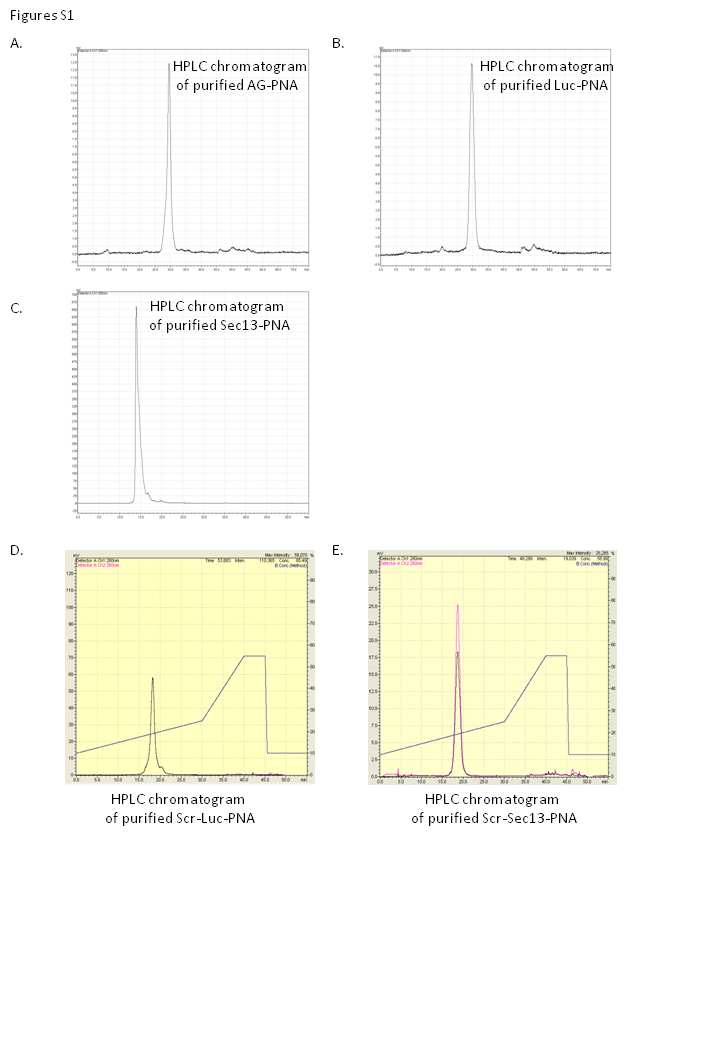

Supplement: Figures S1 — HPLC chromatograms of PNA sequences. PNAs were HPLC purified on a Luna C18 phenomenex column (10 microns, 250×21.2 mm), monitored at 260 nm, using the following eluents: 0.1% TFA in water and acetonitrile; 10-25% gradient of acetonitrile in 30 minutes. (TIF) [file pone.0086802.s001.tif]

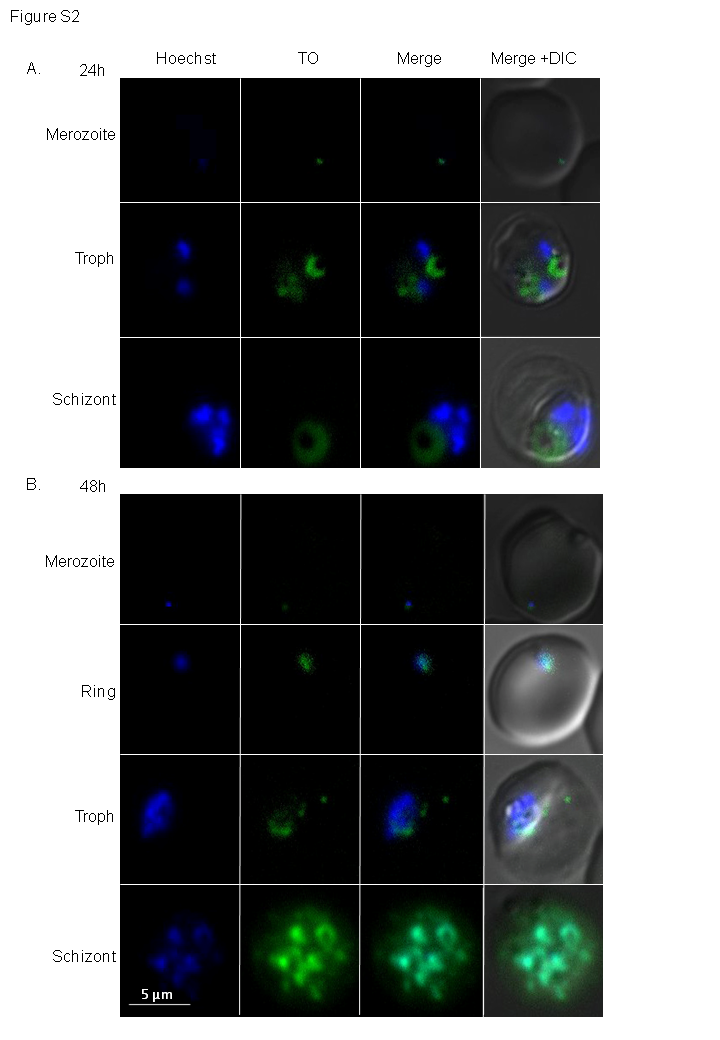

Supplement: Figure S2 — LucPNAs in targeted to intraerythrocytic parasites 24h and 48h post incubation. (A), 24h post incubation with 1.2 µM LucPNA the molecules could already be observed in various stages of development particularly in the FV. We could not detect fluorescent signal in ring stages at this time point. (B), 48h after incubation with 1.2 µM LucPNA the molecules already reach parasites nuclei in various stages of development. Images were taken using exposure time of at least 960ms. (TIF) [file pone.0086802.s002.tif]
